# Vitamin D supplementation improves SIRT1, Irisin, and glucose indices in overweight or obese type 2 diabetic patients: a double-blind randomized placebo-controlled clinical trial

**DOI:** 10.1186/s12875-020-1096-3

**Published:** 2020-02-07

**Authors:** Peivasteh Safarpour, Milad Daneshi-Maskooni, Mohammadreza Vafa, Mitra Nourbakhsh, Leila Janani, Mohsen Maddah, Fatemeh-Sadat Amiri, Fereshteh Mohammadi, Homa Sadeghi

**Affiliations:** 1 Department of Nutrition, School of Public Health, Iran University of Medical Sciences, Tehran, Iran; 2 Department of Nutrition, School of Medicine, Jiroft University of Medical Sciences, Jiroft, Kerman, Iran; 3 Department of Biochemistry, School of Medicine, Iran University of Medical Sciences, Tehran, Iran; 4 Department of Biostatistics, School of Public Health, Preventive Medicine and Public Health Research Center, Iran University of Medical Sciences, Tehran, Iran; 5 Department of Medicine, School of Medicine, Guilan University of Medical Sciences, Guilan, Iran; 6 Department of Epidemiology, University of Massachusetts Lowell, Lowell, MA USA

**Keywords:** Vitamin D, SIRT1, Irisin, Glucose indices, type 2 diabetes, Overweight/obesity

## Abstract

**Background:**

Vitamin D (VD) may increase sirtuin 1 (SIRT1) and subsequently PPAR-γ coactivator 1α (PGC-1α) and irisin levels and these improvements may reduce insulin resistance (IR). The aim was to assess the effects of vitamin D supplementation on SIRT1, irisin, and IR in overweight/obese type 2 diabetes (T2D) patients.

**Methods:**

Ninety T2D males and females were recruited as a clinical trial study (mean of age and body mass index (BMI) of intervention and placebo groups were 50.05 ± 10.17 and 50.36 ± 10.2 yrs. and 31.37 ± 3.4 and 30.43 ± 3.2 kg/m^2^, respectively). The inclusion criteria were T2D, VD deficient, BMI > 25 kg/m^2^, and serum HbA1c < 8.5%. The exclusion criteria were using vitamin and mineral supplements, having any acute disease, recent modifying dose or type of drugs. The supplementation was 50,000 IU/week VD or placebo for 8 weeks. The demographic characteristics, anthropometrics, dietary intakes and physical activity status, sun exposure status, fasting blood sugar (FBS) and insulin, glycosylated hemoglobin (HbA1c), irisin, SIRT1, 25-hydroxy D3 (25(OH)VD), homeostasis model assessment of insulin resistance (HOMA-IR), and quantitative insulin sensitivity check index (QUICKI) were determined. The significant *P*-value was ≤0.05.

**Results:**

The increase of serum VD, SIRT1, and irisin in the intervention group was significant (*p* < 0.001). HbA1c was decreased significantly by 1%. The changes in the other glucose indices (FBS, insulin, and IR) were non-significant.

**Conclusions:**

VD supplementation may improve T2D by decreasing HbA1c and increasing SIRT1 and irisin in VD deficient T2D patients. Further trials are suggested.

**Trial registration:**

Iranian Registry of Clinical Trials, IRCT201604202365N11. Registered 21/08/2016, http://en.irct.ir/trial/2019.

## Background

Diabetes mellitus is a metabolic disease characterized by hyperglycemia resulting from defects in insulin secretion or action or both [1]. Obesity is an important risk factor increases cellular oxidative stress, insulin resistance (IR), and pancreatic beta cells malfunction [2]. In addition, vitamin D (VD) deficiency is highly prevalent in many societies and ages. According to Iranian Multi-Centric Osteoporosis Study (IMOS), 2009, 72.1% of men and 75.1% of women had mild to severe VD deficiency. Globally, about 50% of the population are VD deficient. It is estimated that about 1 billion people all over the world are affected by different degrees of vitamin D deficiency [3–6].

According to recent studies, IR as the most important cause of T2D was inversely related to VD [6–8]. Moreover, serum 25-OH VD is significantly lower among diabetic patients compared with non-diabetic ones [9, 10]. Also, the relationship between serum 25-OH VD and insulin secretion was significant according to some studies [11].

The hyperglycemia, hyperlipidemia, and inflammatory cytokines in diabetes can likely increase oxidative stress and decrease silent mating type information regulation 2 homolog 1 (sirtuin 1 [SIRT1]) levels [12].

Sirtuins as class III histone deacetylases are categorized into 7 types among which, SIRT1 has been studied more than other categories in humans. SIRT1 may play a role in chronic diseases such as diabetes. It affects glucose metabolism in the liver, pancreas, muscle and adipose tissue. The expression and activity of SIRT1 are reduced in hunger, calorie restriction, and some chronic diseases such as diabetes. Accordingly, SIRT1 activation may improve glucose indices and mitochondria function [13–16].

In addition, VD may increase human endothelial SIRT1 level and activity that can be downregulated by Hydrogen Peroxide. Anti-cancer effects of VD are related to VD receptor (VDR) and Forkhead box O (FOXO) protein interaction, phosphorylation stimulation, and SIRT1. The insulin-sensitizing effect of VD is likely in relation to SIRT1 [17].

Irisin is a myokine regulates energy metabolism in exercise and improves insulin resistance [18]. It is secreted in response to Peroxisome Proliferator-Activated Receptor (PPAR)-γ Coactivator (PGC-1α), and low irisin levels may decrease expression and activation of PGC-1α. Also, T2D patients have low serum levels of irisin and PGC-1α [19–22].

**Fig. 1:**
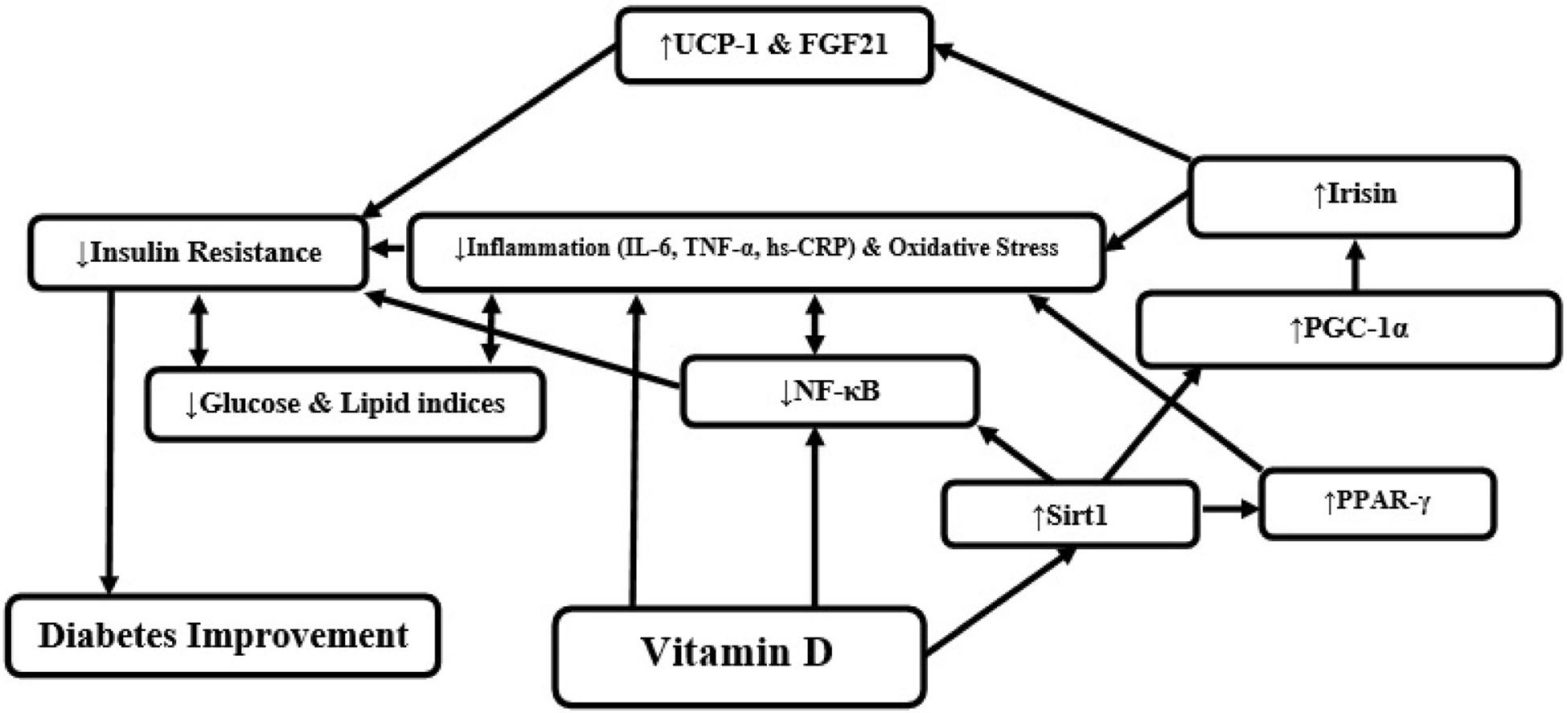
A schematic for the relationship of vitamin D (VD), sirtuin1 (SIRT1), PPAR-gamma coactivator 1-alpha (PGC-1α), insulin resistance (IR), Irisin, and diabetes. (UCP1: Uncoupling Protein 1; FGF21: Fibroblast Growth Factor 21; PPAR-γ: Peroxisome Proliferation Activated Receptor-Gamma; IL-6: Interleukin 6; TNF-α: Tumour Necrosis Factor-Alpha; hs-CRP: high-sensitivity C-Reactive Protein; NF-κB: Nuclear Factor kappa-light-chain-enhancer of activated B cells)

According to the separate studies, VD may increase SIRT1, and subsequently PGC-1α and Irisin. These changes can improve insulin resistance. Thus, VD may decrease insulin resistance by increasing SIRT1 and Irisin (Fig. 1). So, this trial was planned to assess the effects of vitamin D on serum glucose, SIRT1, and Irisin levels in overweight/obese T2D patients.

## Methods

### Study design

This double-blinded randomized clinical trial was conducted on 90 obese T2D patients at Shahid Beheshti Diabetes Clinic, Bandar-E-Anzali, Gilan, Iran. The study lasted from the beginning of autumn 2016 to the end of autumn 2016.

The participants signed a “written informed consent form” at the beginning. Ninety patients were randomly divided into placebo or intervention groups with a 1:1 ratio. The intervention group took 8 VD pearls (50,000 IU/week, Zahravi Co®), and the placebo group took similar pearls containing oral paraffin without VD (50,000 IU/week, Zahravi Co®). The duration of the intervention was 8 weeks. During the intervention, the compliance status including follow-up, side effects, and complications was weekly checked by calling. At the end, the number of the used pearls, returned blisters, and packs were recorded.

**Inclusion** criteria were 25–65 years old, having T2D, serum HbA1c < 8.5%, 25 kg/m2 ≤ BMI, and the written informed consent form. **Exclusion** criteria were the inability to cooperating, acute diseases influencing the intervention, intake of the antioxidant and multivitamin-mineral supplements during the past 3-months so far, changing the type and dose of T2D medications, and taking less than about 90% of the study’s supplements.

The outcomes were serum 25-OH VD, SIRT1, Irisin, HbA1c, IR indexes, FBS, and serum insulin.

### Measures

At the beginning and end, the demographic characteristics, anthropometrics, and dietary intakes by two 24-h food recall (a weekend and a working day) were determined. Physical activity was measured by a short form of the international physical activity questionnaire (IPAQ) [23]. The sun exposure status was assessed using a valid questionnaire [24]. At the beginning and end, the blood taking from the brachial vein for measuring the serum factors was done. Serum glucose was measured by the *Olympus*® device and *Delta*® kits.

The glycosylated hemoglobin was determined using *Nycocard*® kits in *Nyco Card Reader*® (Made in Norway). Insulin was measured by the *Cobas e 411*® device and *Roche*® kit. Irisin was measured by *Zellbio GmbH kit Cat.No: ZB-13253 J-H9648 (Germany)*®, with a normal range of 2–80 ng/ml and sensitivity of 0.1 ng/ml. SIRT1 was measured by *Zellbio GmbH* kit *Cat.No: ZB-12557 J-H9648(Germany)*®, with a normal range of 5–160 ng/ml and sensitivity of 1 ng/ml. ELISA method*; ELISA reader (Model: Tecan A-5082 Made in Austria*®*)*.

25-OH VD was measured by 25-(OH) D_3_ quantitative diagnostic kit (*Immuno Diagnostic Systems (IDS), UK)*®, which is an Enzyme Immunoassay kit with a sensitivity of 5 nmol/L. The ELISA method and ELISA reader *(Model: Tecan A-5082 Made in Austria*®*)* were used for Irisin, SIRT1, and 25-OH D3. The insulin resistance was determined by HOMA-IR and QUICKI indexes [25, 26] as the following formula:

$$ Insulin\ resistance=\left[ fasting\ glucose\left( mmol/L\right)\times fasting\ insulin\ \left(\mu IU/ ml\right)\right]/22.5 $$

$$ Insulin\ Sensitivity=1\div \left[\mathit{\log}\  fasting\ glucose\ \left( mmol/l\right)+\mathit{\log}\  fasting\ insulin\ \left(\mu IU/ ml\right)\right] $$

### Statistical analysis

According to the *Baziar* et al study with 1.33 mean difference of HOMA-IR, 1.18 standard deviation for vitamin D group, and 2.84 standard deviations for placebo group [27], the sample size was calculated 45 peoples in any groups using G-Power software and considering α = 5%, β = 20%, and dropout percentage = 12.5%.

**Fig. 2:**
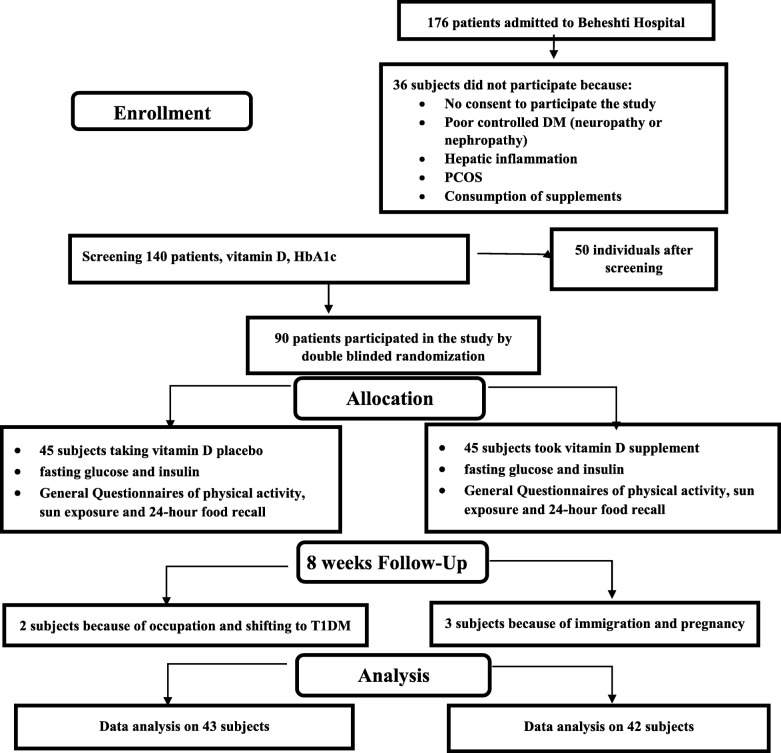
Flow diagram of participants with type 2 diabetes and overweight/obesity grade 1

Randomization was done using 23 quadratic blocks and www.sealedenvelope.com. The blinding was according to unique codes produced by software for each subject. Non-parametric, t-test, Mann-Whitney, Chi-square, and ANCOVA tests and SPSS_16_ statistical software were used for the analyses. The final analysis model was adjusted for the baseline values as potential confounders. *P*-value < 0.05 was statistically considered significant. The dietary intakes were analyzed by the Nutritionist_(IV)_ software. The modified intention-to-treat (m-ITT) method [28] was used for the missing data (3 participants in the intervention group and 2 ones in the placebo group) (Fig. 2).

This clinical trial was approved by the ethics committee of Iran University of Medical Sciences (IUMS) (Code: IR.IUMS.REC.1395.9223475201), and Iranian Registry of Clinical Trials (IRCT) (Registration No: IRCT201604202365N11). An informed consent form (in Persian) obtained from all the participants. Participation was free, and a participant could withdraw at whatever point the person feels he/she was unable to continue. The dose of VD was for insufficient VD status that side-effects had not been reported previously [29]. All of the ethical codes were according to the Helsinki agreement [30]. The personal information of participants was kept secret before, during, and after the trial. After measurements, at the end, a VD supplement was given to the placebo group according to the protocol.

## Results

According to the flow diagram of participants, a total of 176 T2D patients referred to the diabetes clinic were screened (informed consent, medical history, questionnaire), of whom 86 patients did not meet the eligibility criteria. Ninety participants were randomized that 5 patients did not complete the study (for personal reasons; VD group *n* = 3; placebo *n* = 2). Thus 85 participants completed the trial (VD group *n* = 42; placebo *n* = 43) and the data of them were analyzed (Fig. 2).

Most of the participants were women with type 1 obesity (30 ≤ BMI < 35 [BMI = 34.25 kg/m^2^, weight: 59–115 kg, height: 163–184.5 cm]), the mean age of 50 years (25–65 year), recent diagnosed T2D nearly less than 6 years, and consumption of maximum 2 types of anti-hyperglycemic medications. The baseline characteristics including age, gender, weight, height, BMI, duration of diabetes, type and dose of medications, and employment status were similar between two groups before intervention (Table 1).

**Table 1:** The mean and standard deviation of the baseline characteristics of type 2 diabetes patients

Variable	Category	Group		*P*-value
		Vitamin D (*N* = 42)	Placebo (*N* = 43)	
Age (Y)	–	50.36 (10.2)	50.05 (10.7)	0.892^*^
Weight (kg)	–	77.38 (10.9)	79.27 (11.6)	0.442^*^
Height (cm)	–	159.35 (8.0)	158.77 (7.6)	0.736^*^
BMI (kg/m ^2)^)	–	30.43 (3.2)	31.37 (3.4)	0.194^*^
Disease duration (months)	–	65.52 (38.5)	67.02 (35.4)	0.664^*^
Medications (per day)	Metformin (500 mg/d)	2.11 (1.13)	2.51 (1.26)	0.135^*^
	Glibenclamide (5 mg/d)	1.31 (0.94)	0.95 (0.78)	
Gender	Male	7.00 (16.7)	8.00 (18.6)	0.815**
	Female	35.00 (83.3)	35.00 (81.4)	
Employment status	Unemployed	32.00 (76.2)	32.00 (74.4)	0.850**
	employed	10.00 (23.8)	11.00 (25.6)	

*Independent t-test; **Chi-square test, *Y* Year, *kg* Kilogram, *cm* Centimeter, *BMI* Body mass index, *mg* Milligram

Physical activity status (MET-min/week) was not significantly different between the two groups. Within the placebo group, weight and BMI decreased significantly (*P* < 0.05). However, these differences were not significant between the two groups (*P* > 0.05) (Table 2).

**Table 2:** The mean and standard deviation of physical activity, weight, and BMI in type 2 diabetes patients

Variable		Vitamin D (*N* = 42)	Placebo (*N* = 43)	*P*-value^*^
Physical activity (MET. Minutes/week)	Baseline	685.50 (99–34,755)	462.00 (99–28,560)	0.346
	After intervention	693.00 (99–33,831)	462.00 (99–28,659)	0.333
	*P*-value^**^	0.104	0.811	
Weight (kg)	Baseline	77.38 (10.92)	79.27 (11.67)	0.442
	After intervention	77.13 (10.81)	78.77 (11.53)	0.502
	*P*-value^**^	0.076	**0.011**	
BMI (kg/m^2^)	Baseline	30.43 (3.23)	31.37 (3.40)	0.194
	After intervention	30.34 (3.32)	31.18 (3.42)	0.254
	*P*-value^**^	0.097	**0.009**	

*Independent t-test, **Paired t-test, *kg* Kilogram, *cm* Centimeter, *BMI* Body mass index, *mg* Milligram, *MET* Metabolic equivalent

Energy, macronutrients, micronutrients, and antioxidant intakes were not significantly different within and between groups (Tables 3 and 4). Within the VD group, zinc intake increased significantly (P < 0.05), but within the placebo group, zinc intake decreased significantly (P < 0.05). However, this difference was not significant between the two groups (P > 0.05, Table 4).

**Table 3:** The mean and standard deviation of energy intake, macronutrients, and micronutrients before and after intervention in type 2 diabetes patients

		Vitamin D (*N* = 42)	Placebo (*N* = 43)	*P*-value^*^
Energy (kcal/day)	Baseline	1638.59 (377.82)	1578.38 (399.64)	0.478
	After intervention	1701.36 (485.61)	1564.68 (392.75)	0.157
	*P*-value^**^	0.381	0.871	
Protein (g/day)	Baseline	82.27 (23.34)	78.79 (31.82)	0.568
	After intervention	89.31 (35.83)	80.31 (32.16)	0.226
	*P*-value^**^	0.190	0.823	
Carbohydrate (g/day)	Baseline	232.69 (67.62)	220.85 (54.71)	0.377
	After intervention	231.41 (62.34)	214.21 (51.19)	0.168
	*P*-value^**^	0.891	0.522	
Total fat (g/day)	Baseline	47.61 (11.25)	46.83 (12.22)	0.760
	After intervention	51.31 (17.33)	47.41 (14.13)	0.258
	*P*-value^**^	0.199	0.830	
SFA (g/day)	Baseline	16.10 (4.58)	15.35 (3.75)	0.413
	After intervention	16.01 (6.10)	15.74 (4.54)	0.819
	*P*-value^**^	0.913	0.639	
MUFA (g/day)	Baseline	16.06 (3.85)	15.59 (5.07)	0.636
	After intervention	17.13 (5.85)	15.56 (4.86)	0.182
	*P*-value^**^	0.265	0.976	
PUFA (g/day)	Baseline	9.98 (3.04)	10.04 (3.45)	0.836
	After intervention	10.49 (2.47)	9.97 (3.86)	0.480
	*P*-value^**^	0.365	0.919	
Calcium (mg/day)	Baseline	835.33 (279.75)	838.41 (301.51)	0.961
	After intervention	804.44 (324.89)	833.63 (292.95)	0.665
	*P*-value^**^	0.487	0.929	
Phosphorus (mg/day)	Baseline	1444.29 (402.18)	1364.80 (372.84)	0.347
	After intervention	1459.15 (467.88)	1355.23 (388.99)	0.268
	*P*-value^**^	0.814	0.897	
Vitamin D (μg/day)	Baseline	1.73 (1.28)	1.84 (1.41)	0.710
	After intervention	1.58 (1.18)	1.64 (1.22)	0.835
	*P*-value^**^	0.386	0.334	
Total fiber (g/day)	Baseline	29.23 (10.67)	26.58 (7.56)	0.192
	After intervention	29.96 (8.25)	25.91 (9.05)	0.578
	*P*-value^**^	0.127	0.603	

*Independent t-test, **Paired t-test, *SFA* Saturated fatty acid, *MUFA* Mono unsaturated fatty acid, *PUFA* Poly unsaturated fatty acid

**Table 4:** The mean and standard deviation of the dietary antioxidants before and after intervention in type 2 diabetes patients

		Vitamin D (*N* = 42)		Placebo (*N* = 43)		*P*-value^*^
Beta-Carotene (micg/day)	Baseline	3780.76	3117.63	5440.75	6623.31	0.242
	After intervention	4379.33	4073.18	4419.51	4235.22	
	*P*-value^**^	0.362		0.963		
Vitamin A (micg/day)	Baseline	878.74	663.17	893.10	723.97	0.952
	After intervention	757.20	433.10	781.86	489.78	
	*P*-value^**^	0.059		0.947		
Vitamin E (mg/day)	Baseline	15.79	4.15	15.96	4.63	0.951
	After intervention	15.42	4.18	15.67	4.13	
	*P*-value^**^	0.882		0.522		
Vitamin C (mg/day)	Baseline	122.87	81.12	139.18	108.88	0.596
	After intervention	120.19	74.06	123.56	76.57	
	*P*-value^**^	0.101		0.528		
Selenium (mg/day)	Baseline	238.09	54.43	235.76	88.75	0.386
	After intervention	213.06	61.64	230.75	83.54	
	*P*-value^**^	0.696		0.887		
Zinc (mg/day)	Baseline	20.61	5.82	20.46	5.85	0.306
	After intervention	20.96	6.40	19.39	6.05	
	*P*-value^**^	0.003		0.005		
Manganese (mg/day)	Baseline	12.57	3.61	12.83	5.54	0.381
	After intervention	11.48	4.04	12.79	4.62	
	*P*-value^**^	0.240		0.052		
Copper (micg/day)	Baseline	2.79	0.75	2.67	1.05	0.268
	After intervention	2.54	0.75	2.73	0.93	
	*P*-value^**^	0.364		0.988		
Magnesium (mg/day)	Baseline	717.30	168.48	725.50	247.19	0.461
	After intervention	680.08	191.00	732.94	224.49	
	*P*-value^**^	0.695		0.284		

*Independent t-test, **Paired t-test

According to the final analysis model, the differences of serum VD, SIRT1, Irisin, and HbA1c were significant within and between groups (P < 0.05). Serum VD level was doubled compared to the baseline level, and HbA1c decreased by 1% among the VD group (Table 5). Within the VD group, irisin and SIRT1 increased significantly (P < 0.05). The differences in fasting glucose and insulin, HOMA-IR, and QUICKI were not significant between the two groups (Table 6). The sun-exposure status was not significantly different within and between groups.

**Table 5:** The mean and standard deviation of the primary variable before and after intervention in type 2 diabetes patients

		Vitamin D (*N* = 42)	Placebo (*N* = 43)	crude *P*-value^*^	Adjusted *P*-value^***^
VitaminD (ng/ml)	Baseline	17.24 (7.83)	17.56 (7.82)	0.853	< 0.001
	After intervention	38.86 (10.76)	14.79 (7.08)	< 0.001	
	*P*-value^**^	< 0.001	< 0.001		
HbA1c (%)	Baseline	7.51 (0.87)	7.15 (1.12)	0.103	< 0.001
	After intervention	6.76 (0.98)	7.21 (1.11)	0.051	
	*P*-value^**^	< 0.001	0.657		
SIRT1 (ng/ml)	Baseline	24.94 (4.38)	25.57 (3.37)	0.455	< 0.001
	After intervention	27.90 (5.58)	23.40 (3.88)	< 0.001	
	*P*-value^**^	< 0.001	< 0.001		
Irisin (ng/ml)	Baseline	18.15 (5.96)	20.13 (7.17)	0.171	
	After intervention	21.99 (5.93)	14.98 (2.76)	< 0.001	< 0.001
	*P*-value^**^	< 0.001	< 0.001		
HOMA-IR (N)	Baseline	5.36 (3.23)	5.83 (3.09)	0.499	0.421
	After intervention	6.44 (3.73)	7.39 (4.58)	0.295	
	*P*-value^**^	0.006	0.003		
QUICKI (N)	Baseline	0.76 (0.08)	0.73 (0.06)	0.149	0.645
	After intervention	0.73 (0.07)	0.71 (0.07)	0.217	
	*P*-value^**^	0.005	0.003		

*Independent t-test, **Paired t-test, ***ANCOVA

**Table 6:** The mean and standard deviation of the secondary variable before and after intervention in type 2 diabetes patients

		Vitamin D (*N* = 42)	Placebo (*N* = 43)	*P*-value^*^	Adjusted *P*-value^***^
FBS (mg/dl)	Baseline	172.48 (62.16)	178.28 (71.74)	0.629	0.130
	After intervention	175.52 (65.44)	162.72 (61.36)	0.355	
	*P*-value^**^	0.742	0.053		
Insulin (micIU/ml)	Baseline	12.64 (5.94)	14.09 (6.43)	0.284	0.101
	After intervention	15.31 (7.24)	18.77 (9.02)	0.055	
	*P*-value^**^	0.002	< 0.001		

*Independent t-test, **Paired t-test, ***ANCOVA

### Safety

The patients reported no side effects associated with treatment.

## Discussion

Vitamin D supplementation (50,000 IU/week) for 8 weeks increased serum VD levels in T2D patients with VD deficiency. Compared to placebo, VD increased SIRT1 and Irisin and decreased HbA1c by 1% significantly. The differences in fasting insulin and glucose, HOMA-IR, and QUICKI were not significant. Within the placebo group, serum VD levels decreased significantly.

The results of some studies of the effects of VD supplementation on diabetes showed controversy that the following is referred to some of them.

In some studies, VD effects on insulin and glucose levels of diabetic patients were similar to our trial [31–36].

In line with our results, *Polidoro* et al*, 2013* reported that SIRT1 and Irisin levels increased in the VD group and decreased in the placebo group significantly [17]. The decreases can likely be attributed to progressing VD deficiency in the placebo group after 2 months.

In *Anastasilakis* et al*, 2013* study, irisin levels after osteoporotic fractures in postmenopausal women with low bone mineral density for 3 months didn’t show a significant correlation with serum 25(OH)VD levels. The decrease of Irisin was related to physical inactivity and a sedentary lifestyle during fracture [37]. In another study on healthy peoples, a one-time injection of 100,000 IU VD increased serum 25(OH)VD levels and the changes of serum irisin were non-significant. Only in one subject out of 28, the serum irisin increased by three times; which may be related to genetic variations in response to the intervention [38].

A study in 2017 showed serum irisin levels were decreased after 2 weeks’ mountain climbing. The possible reasons were exposure to hypoxia, the energy-related mechanisms, and the revitalization of musculoskeletal cells in response to hypoxia [39].

The changes in physical activity in our study were not significant. Accordingly, the improvement of serum irisin levels in the VD group was independent of physical activity levels. On the other hand, the decrease and increase of irisin can be related to serum VD levels or the dietary intake of VD in T2D patients.

According to the different studies, the effects of VD on glucose indices are controversial.

Similar to our study, two separate studies with 50,000 IU/d VD didn’t find significant effects on FBS levels [33, 34]. However, in two other studies, the increased serum VD levels were significantly related to FBS [40, 41]. The likely reasons may be the dietary patterns, timing or type of hyperglycemia medications, and the genetic differences of the populations.

In contrast, some studies of VD effects reported a significant improvement in fasting serum insulin [34, 42–44]. The potential mechanisms may be the presence of VD receptors (VDRs) in pancreas cells and expression of 1-alpha-hydroxylase in them [37] and the existence of some elements on human insulin promotors that respond to VD and can activate insulin gene transcription [38–40].

In line with this trial, the different studies showed a significant decrease in HbA1c of diabetics’ patients [33, 34, 43, 44]. However, the effect of VD on HbA1c was non-significant in two separate studies [45, 46]. The reasons may be the unadjusted potential confounders and the different design of these studies. The potential mechanisms are unknown.

No significant changes in insulin resistance and sensitivity of our trial were similar to *Jorde* et al*, 2010* study [40], and *SUNNY* trial [34]. However, some studies reported a significant improvement in HOMA-IR or QUICKI with vitamin D [43, 44, 47]. The reason may be changing fasting serum glucose and insulin levels after the intervention. Vitamin D may decrease blood glucose by increasing insulin sensitivity, glucose uptake of peripheral tissues, and glycogen synthesis in liver [14]. The existence of VDR and expression of 1-α-hydroxylase in pancreatic cells may be a probable mechanism of the VD effect on insulin secretion [48]. Also, response elements to VD in human insulin promoters may activate insulin gene transcription [15, 16].

The lower serum 25(OH)-VD level is associated with abdominal and peripheral obesity, physical inactivity, smoking, alcohol intake history, and the lower dietary intake of fish. In addition, obesity and physical inactivity are important risk factors for T2D. However, in this trial, differences in physical activity levels were non-significant. Serum VD levels can be directly related to central adiposity [49] wasn’t measured in our participants. Accordingly, the relationship between Vitamin D and glucose profiles can also be interpreted.

Vitamin D can increase insulin receptor gene expression in beta cells, glucose transport in the intestine [50], and intestinal calcium absorption may serve as stimuli for insulin release. In addition, some VDRs on beta cells can turn 25(OH)D3 (calcidiol) into the active VD [51, 52].

The more reasons for the differences between our trial and other studies may be unadjusted confounders, the different designs, the variety of dietary patterns, and the dose or timing of hyperglycemia medications.

This study has several strengths. First, the double-blinded stratified blocked randomization design; Second, considering multiple eligibility criteria; Third, the determining of dietary intakes and physical activity status and adjusting the statistical analysis for them and other potential confounders; Fourth, considering control group. These strengths are likely preferable in comparison with the few other clinical trials that have evaluated the effects of VD in T2D.

However, our study had some limitations. First, the sample size was small; Second, the intervention duration was likely short to understand the real effects of VD supplementation on the different markers in T2D patients; Third, disregarding non-obese patients. Fourth, self-reporting of diet and physical activity; Fifth, failure to measuring serum parathyroid hormone (PTH), Apo-lipoprotein A1, and the more sensitive inflammatory factors; Sixth, 24-h food recall is not a good index for assessing the usual food intake; Seventh, failure to measuring body composition. Even so, this study is the first trial of VD effects on serum SIRT1 and irisin levels in overweight/obese T2D patients.

We suggest similar trials measuring adipose tissue distribution and considering more potential confounders and the longer intervention period.

## Conclusion

Vitamin D supplementation improves serum 25-(OH) VD, SIRT1, irisin, and glycosylated hemoglobin in VD deficient T2D patients. The decrease of HbA1c may be related to the increased serum VD and irisin levels. Further trials are suggested.

## Supplementary information

**Additional file 1: Table S1.** The measurement tools and descriptions of companies.

## Data Availability

The datasets used and/or analyzed during the current study are available from the corresponding author on a reasonable request.

## Abbreviations

ANCOVA: Analysis of covariance
BMI: Body mass index
ELISA: Enzyme-linked immunosorbent assay
FBS: Fasting blood sugar
FGF21: Fibroblast Growth Factor 21
FOXO: Forkhead box O
HbA1c: Glycosylated hemoglobin
HOMA-IR: Homeostasis model assessment-insulin resistance
hs-CRP: High-sensitivity C-Reactive Protein
IL-6: Interleukin 6
IMOS: Iranian Multi-Centric Osteoporosis Study
IPAQ: International physical activity questionnaire
IR: Insulin resistance
ITT: Intention to treat
NF-κB: Nuclear Factor kappa-light-chain-enhancer of activated B cells
PGC-1α: PPAR-γ coactivator-1 alpha
PPAR: Peroxisome proliferation activated receptor
PPAR-γ: Peroxisome Proliferation Activated Receptor-Gamma
QUICKI: Quantitative insulin sensitivity check index
SIRT1: Sirtuin-1
T2D: Type 2 diabetes
TNF-α: Tumour Necrosis Factor-Alpha
UCP1: Uncoupling Protein 1
VD: Vitamin D
VDR: Vitamin D receptor
