# Supplementary material for: Vitamin D supplementation improves SIRT1, Irisin, and glucose indices in overweight or obese type 2 diabetic patients: a double-blind randomized placebo-controlled clinical trial
Source: BMC Fam Pract. 2020 Feb 7;21:26. doi: 10.1186/s12875-020-1096-3 (PMC7007689; doi:10.1186/s12875-020-1096-3)
Supplement: Supplementary file 1 — Additional file 1: Table S1. The measurement tools and descriptions of companies. [file 12875_2020_1096_MOESM1_ESM.docx]

**Table S1.** The measurement tools and descriptions of companies

| **Items** | **Tools** | **Company** |
| --- | --- | --- |
|  |  |  |
| Glucose | Olympus® device, Delta® kits | Delta Kits |
| Glycosylated Hemoglobin | Nycocard® kits, Nyco Card Reader® | NPT Co.Ltd. |
| Insulin | Cobas e 411® device, Roche® kit | Roche Co |
| Irisin | Zellbio GmbH kit Cat.No: ZB-13253J-H9648 (Germany)®, ELISA reader (Model: Tecan A-5082 Made in Austria®) | ZellBio GmbH |
| Sirtuin1 | Zellbio GmbH kit Cat.No: ZB-12557J-H9648(Germany)®, ELISA reader (Model: Tecan A-5082 Made in Austria®) | ZellBio GmbH |
| 25-(OH) D3 | (Immuno Diagnostic Systems [IDS] Kit, UK)®, ELISA reader (Model: Tecan A-5082 Made in Austria®) | Immunodiagnostic Systems Holdings PLC |
